# Attributes of Yellow Traps Affecting Attraction of *Diaphorina citri* (Hemiptera: Liviidae)

**DOI:** 10.3390/insects11070452

**Published:** 2020-07-16

**Authors:** Sandra A. Allan, Justin George, Lukasz L. Stelinski, Stephen L. Lapointe

**Affiliations:** 1 Center for Medical, Agricultural and Agricultural Entomology, Agricultural Research Service, United States Department of Agriculture, Gainesville, FL 32608, USA; 2 US Horticultural Research Laboratory, Agricultural Research Service, United States Department of Agriculture, 2001 South Rock Road, Fort Pierce, FL 34945, USA; georgejustine@gmail.com (J.G.); stephenlapointe@bellsouth.net (S.L.L.); 3 Entomology and Nematology Department, Citrus Research and Education Center, University of Florida, 700 Experiment Station Rd, Lake Alfred, FL 33850, USA; stelinski@ufl.edu

**Keywords:** psyllid, vision, traps, color, yellow, surveillance, UV reflectance

## Abstract

Laboratory assays were conducted to evaluate responses of *Diaphorina citri* to various aspects of visual cues associated with traps in an effort to improve trap effectiveness. Addition of white or UV violet but not yellow light-emitting diodes (LEDs) increased attraction to standard yellow adhesive traps moderately (11–17%), with no difference in attraction between white or UV violet LEDs. Addition of a black border on yellow traps enhanced collections. However, there were no differences between attraction to black patterns on traps. Comparisons were made between different commercial paints, some with UV-reflecting properties or fluorescence. A yellow paint with UV reflectance, used for painting bird decoys (decoy yellow), was more attractive than the standard yellow Olson sticky trap. Addition of white or green pigment to increase intensity or enhance green reflectance, respectively, did not increase attraction. Alteration of reflectance of Olson traps with addition of UV-reflecting or fluorescent pigments did not enhance attraction of *D. citri*. In field comparisons, decoy yellow and fluorescent yellow sticky traps were more attractive to *D. citri* than Olson yellow.

## 1. Introduction

Huanglongbing or citrus greening disease severely limits citrus production in Florida and throughout the world. The phloem-limited bacterial pathogen *Candidatus* Liberibacter asiaticus (CLas) is transmitted by the Asian citrus psyllid, *Diaphorina citri* Kuwayama (Hemiptera: Liviidae) [1,2]. Intensive and area-wide application of broad-spectrum chemical insecticides has been the primary means attempted to control the vector and the disease to date [3,4]. Surveillance based on attraction of *D. citri* to yellow sticky cards helps guide management decisions [5,6]. While these traps are a standard in the industry, they have limited ability to detect low levels of infestation [7]. These studies were initiated to examine visual properties in an attempt to produce a better trap and possibly to contribute to the production of an attract-and-kill device.

There is strong evidence that adult *D. citri* primarily use visual cues to orient at a distance to, and gustatory cues to settle on, host plants [8,9,10]. Olfactory orientation has not been conclusively demonstrated despite attempts to do so [8,10,11,12,13,14]. Vision in *D. citri* is mediated by photoreceptors sensitive to ultraviolet (UV), blue, and green/yellow portions of the visual spectrum [15]. Similar to many phytophagous insects, visual attraction of *D. citri* is strongest to yellow and yellow/green reflective targets as demonstrated in the laboratory and under field conditions [7,16]. Laboratory studies have also demonstrated that *D. citri* is attracted to UV, yellow and green light-emitting diodes (LEDs) [17]. Phototactic responses by *D. citri* were greater to short wavelengths (UV, violet) compared with longer wavelengths (green, yellow, orange) [18]. The removal of UV wavelengths from green or yellow light significantly reduced attraction, indicating that UV wavelengths may play a role in attraction to long wavelengths [19]. Addition of a UV-reflecting pigment, magnesium oxide (MgO), increased attraction to yellow traps in the laboratory and field and enhanced probing behavior when combined in a phagostimulant blend [9]. Female *D. citri* prefer emergent citrus leaves before they are fully expanded for feeding and oviposition and these leaves are characteristically high-intensity yellow/green [16,18]. The most effective sticky traps reported to date for collection of *D. citri* are yellow or yellow/green cards that closely mimic reflectance of new flush tissue [16]. Yellow/green traps attractive to many leaf- and flower-feeding insects are considered by some as supernormal stimuli [20,21].

Characteristics of visual signals that affect attraction of insects to a target include hue, saturation, brightness, translucency, and polarization [20,21,22,23], with some possible effects from fluorescence or iridescence [23,24,25]. The size and shape of targets [26,27,28], as well as contrast to background [29,30,31,32], are important features affecting attraction of a wide range of insect species to monitoring traps. The presence of black edges or patterns on visual targets may increase insect captures on traps, as seen with house flies [33,34] and whiteflies [23]. The length of contrasting edges on trap surfaces contributes to attraction of aphids and thrips [26,35]. Target intensity and contrast against background were shown to be important for orientation and landing of a butterfly [31]. 

Greater understanding of factors that mediate the behavioral response of *D. citri* to visual cues should improve effectiveness of monitoring traps and contribute to development of attract-and-kill devices. In this study, we evaluated aspects of yellow traps that may be manipulated to improve attraction of *D. citri* to visual targets. Experiments included evaluations of light-emitting diodes (LEDs), black patterns, UV reflectance, fluorescence and paint mixtures on attraction.

## 2. Materials and Methods

### 2.1. Psyllids

Insects were reared on *Murraya paniculata* (L.) Jack (Rutaceae) in a greenhouse at the Center for Medical, Agricultural and Agricultural Entomology (CMAVE), Gainesville, FL, at 29 ± 2 °C, under natural and metal halide lighting, with a photoperiod of 16:8 (L:D). Plants were watered twice weekly and fertilized monthly with Miracle-Gro^®^ (The Scott’s Company, Marysville, OH, USA). Rearing plants and randomly selected psyllids were determined to be free of CLas infection using PCR [36]. Adult psyllids were collected from rearing cages by aspirator and thus were of mixed age and sex.

### 2.2. Laboratory Behavioral Assays

The response of adult *D. citri* to visual stimuli was evaluated with sticky-surfaced colored traps in a laboratory. All traps were square (10 × 10 cm) and modified from commercial sticky traps or prepared by application of specific coatings. The surface was covered by a clear double-sided hot melt, pressure-sensitive adhesive panel (Alpha Scents, West Lynn, OR) or sticky adhesive (Tangle-Trap^®^, Grand Rapids, MI). Psyllid response was evaluated with either 2- or multi-choice assays in white screened cages (47.5 × 47.5 × 47.5 cm or 47.5 × 47.5 × 93 cm, respectively), with clear plastic tops (MegaView Science, Taichung, Taiwan) that allowed UV light transmission. Cages were placed within an arena enclosed with white cloth, illuminated with 250 watt metal halide lights which provide UV and visible light [19] and maintained at 28 ± 1 °C. Bias was minimized by rotating the position of treatments for each replicate. Sticky traps were attached to one wall of the cage at equal distance from each other prior to release of 40 or 90 adult *D. citri* into each cage for 2- or multi- choice assays, respectively, at 8:00 h. Traps were removed from the cages 6 h later. Live psyllids not adhered to sticky traps were collected and considered non-responders. Traps were examined and psyllids sexed and counted under a microscope.

#### 2.2.1. LEDs

White, UV violet and yellow LEDs (Intelliflame, Waterford, WI, USA) were tested in conjunction with commercial Olson yellow sticky traps (10 × 10 cm, Olson Products, Medina, OH, USA). LED bulbs were inserted through holes in the center of traps with the battery assembly behind the trap. Assays were conducted as 2-choice paired tests, either comparing Olson yellow traps with and without an LED or between traps with either a UV or white LED. Tests were conducted for 20 replicates.

#### 2.2.2. Black Patterns

**Figure 1:**
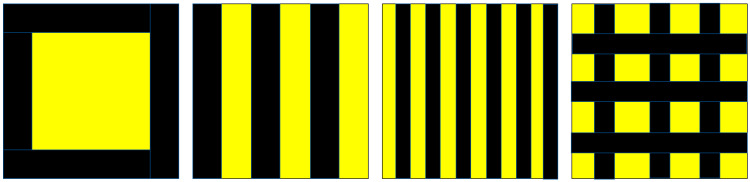
Diagrammatic representation of the yellow and black patterned traps used for evaluation of attraction of *D. citri*. Half of the surface area of each trap was yellow.

Traps were prepared to study attraction of adult *D. citri* to commercial yellow traps modified with black areas. An initial comparison was performed to determine whether addition of black edges increased collection of psyllids. This was conducted as a 2-choice assay comparing responses to an Olson yellow sticky trap (10.16 × 10.16 cm) to a similar-sized trap but placed against a black border. The black-bordered traps were made from black poster board cut into squares (11.16 × 11.16 cm), overlaid with a yellow square cut from Olson yellow traps and covered by a clear double-sided adhesive panel. Assays were replicated 12 times. The second evaluation was to determine whether attraction differed between different black and yellow patterns when the surface area of yellow was the same with each pattern. These traps were 10.16 × 10.16 cm, made similarly from black poster board and covered with clear adhesive panels. Yellow traps with black patterns were made from yellow trap material that was cut into one large square (7.18 × 7.18 cm) and placed in the center of the black square; cut into wide yellow stripes (10.16 cm long × 1.69 cm wide) and placed to produce equal-sized black stripes; cut into narrow stripes (10.16 cm long × 0.85 cm wide) and placed to produce equal-sized black stripes; or cut into 16 small yellow squares (1.79 × 1.79 cm) and placed in 4 rows each with 4 squares (Figure 1). Two layers of yellow were applied to each trap to produce a vivid yellow color. These four configurations of a large square, wide stripes, narrow stripes and small squares provided a total of 28.72, 50.80, 111.76 and 85.92 cm, respectively, of black/yellow edges. Traps were assessed in a 4-choice assay, replicated 12 times. Psyllids adhered to the yellow or black portions of the trap were recorded separately.

#### 2.2.3. UV Reflectance or Fluorescence

Commercially available paints varying in visible and UV reflectance were compared with Olson yellow traps in a series of five experiments. Experiment 1 consisted of a comparison of four white paints that differed in UV reflectance or contained fluorescent pigments: white titanium dioxide acrylic primer (not UV-reflecting), acrylic fluorescent neon white (GloMania, Cottonwood Heights, UT, USA), acrylic fluorescent Wildfire white and UV-reflecting white (ReelWing, Fargo, ND, USA). In experiment 2, UV-reflecting paints designed for bird decoys were compared and included green, iridescent green, white and yellow paints. In experiment 3, responses to purple, red and orange fluorescent paints were compared (Wildfire Inc., Torrance, CA, USA). In experiment 4, comparisons were made between green, orange, purple, white and yellow fluorescent paints (GloMania). As yellow traps were the most attractive, attraction to the standard Olson yellow trap, two fluorescent yellow paints (neon yellow, GloMania and Wildfire yellow, Wildfire, Inc.) and a UV-reflecting yellow paint used for bird decoys (ReelWing) was compared in experiment 5. Paints were applied to acetate sheets previously coated with white titanium dioxide acrylic primer then covered with a clear sticky sheet on one side. Experiments were multi-choice tests with traps placed in a horizontal line on the bottom of the cage with equal spacing between traps. All experiments were replicated 12 times except for the one comparing yellow paints with Olson traps and these were replicated 28 times. Trap positions were randomly assigned and rotated between replicates.

Another series of experiments examined the effect of UV-reflecting or fluorescent pigments applied to Olson yellow traps. In experiment 6, 1.25, 2.50 or 3.75 g of MgO (UV-reflecting pigment) powder (Grace Chemical Co, Curtis Bay, MD, USA) was added to 1 gm of sticky adhesive and applied to Olson yellow traps to examine its effect on *D. citri* attraction. In experiment 7, Olson yellow traps were prepared with a florescent pigment WTR (water-based) (GloMania) that was either yellow or white and traps prepared with MgO were included for comparison. In experiment 8, Olson yellow traps were examined with another fluorescent pigment UVI (Invisible black light neon pigment) (GloMania, Cottonwood Heights, UT, USA) that was either yellow or green in color. These assays were replicated 12 times.

#### 2.2.4. Paint Mixtures

Traps were prepared using paint combinations to evaluate whether increased reflectance would increase attraction to yellow. In experiment 9, mixtures of decoy paints were prepared based on% weight composition by combining yellow and white paints (100:0, 90:10, 80:20 or 70:30 yellow:white). As lime green has been reported to be attractive to *D. citri* [7,37], mixtures of green and yellow decoy paint (100:0, 90:10, 80:20, 70:30 and 0:100 yellow:green) were also compared in experiment 10. Mixtures of florescent paints were similarly prepared using yellow and green Wildfire paint (100:0, 75:25%, 25:75% and 100:0 yellow:green) in experiment 11. Paint mixture assays were replicated 12 times.

### 2.3. Field Assays

Field evaluations of yellow sticky traps were conducted in a grove of white grapefruit, *Citrus paradise* cv. marsh located at Emerald Groves Inc., Fort Pierce, FL. Test traps consisted of Olson yellow sticky traps, decoy yellow traps and two fluorescent yellow traps (neon yellow and Wildfire yellow). Each trap was paired with a similar-sized standard yellow sticky trap (Great Lakes IPM, Vestaburg, MI, USA) [5] placed 1.2 m apart on the same tree. Each trap type with paired standard was placed at six locations, 15 trees apart (45 m). The number of adult *D. citri* trapped were counted 7 d after deployment on August 2 and again on August 9, 2018. As there were no differences in collections between dates (paired *t*-test, α = 0.05), data were pooled (*n* = 12) and comparisons between the test trap and the standard yellow traps analyzed using paired *t*-tests in JMP statistical program (α = 0.05) (SAS, Cary, NC, USA).

### 2.4. Reflectance Measurements

LED spectral emission was measured with a concave grating spectrometer (UV–VIS Black Comet, StellarNet Inc, Tampa, FL, USA) using quartz light guides. Reflectance from trap surfaces was measured with a deuterium/tungsten/halogen light source (StellarNet, Inc, Tampa, FL, USA), with probe and illumination at a 45° angle to the trap surface. Reflectance measurements were standardized to a white standard (RS-50, StellarNet Inc, Tampa, FL, USA) consisting of Halon (polytetrafluoroethylene powder) that reflected > 98% from 300 to 700 nm and a dark standard (no light). Each surface was measured at 0.5 nm intervals from 300 to 700 nm and then averaged.

### 2.5. Statistical Analysis

For laboratory assays, data analyzed were percentages of psyllids collected on traps (dependent variable). Within each trap collection, there were no differences in collections by sex (paired *t*-test; α = 0.05); therefore, data were pooled by sex. Data were tested for normality (Shapiro–Wilk test) and means were compared by paired *t*-test or analysis of variance (ANOVA) if normal and by Mann–Whitney rank sum test and Kruskal–Wallis ANOVA on ranks, if not normal (α = 0.05). For field tests, data tested were numbers of psyllids collected on traps (dependent variable) and comparisons were made using paired *t*-tests. Analyses were conducted with SigmaStat, San Jose, CA, USA.

## 3. Results

### 3.1. Laboratory Behavioral Assays

#### 3.1.1. LEDs

**Figure 2:**
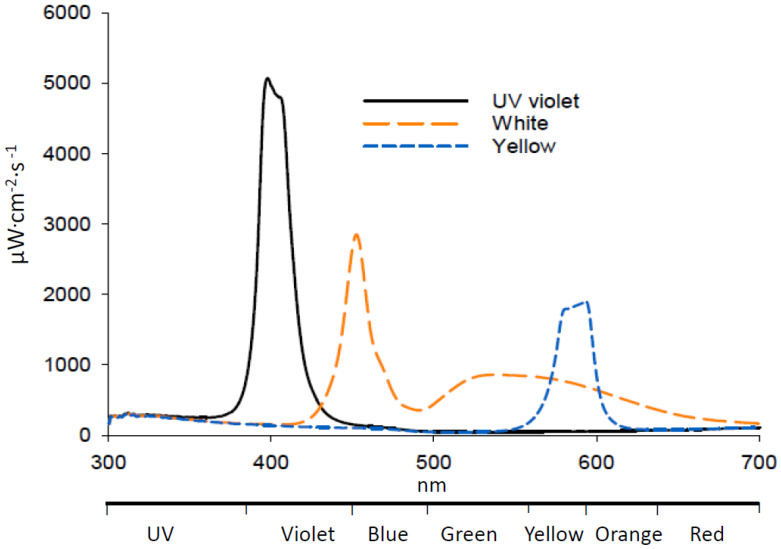
Emission spectra of LEDs used in attraction assays.

White LED emission consisted of major peaks at 453 and 530 nm, with overall intensity between 300 and 700 nm of 2041 µW cm^−2^ s^−1^ at 2 cm from the light source, 15% reflectance from 300 to 400 nm and 85% from 400 to 700 nm (Figure 2). The UV LED emitted in one peak at 400 nm with overall emission of 2237 µW cm^−2^·s^−1^, of which 53% was from 300 and 400 nm and 47% from 400 and 700 nm. The yellow LED had a peak at 594 nm and an overall emission of 849 µW cm^−2^ s ^−1^. Addition of either UV or white LEDs significantly increased collection of adult *D. citri* on Olson sticky traps by 11 and 17%, respectively, compared with a yellow adhesive card with no LED illumination (Table 1). Addition of a yellow LED did not affect trap catch compared with a yellow adhesive card with no LED illumination. There was no significant difference in attraction of *D. citri* to cards illuminated with UV or white LEDs.

#### 3.1.2. Black Patterns

The addition of black edges on yellow traps significantly increased attraction to traps (t = 7.27, df = 22, *p* < 0.001), with yellow traps collecting 24.0 ± 5.1% and black and yellow traps collecting 75.9 ± 5.0% of psyllids. The yellow area on the black and yellow traps was the same as that on the completely yellow control traps but collected significantly more psyllids (50.7 ± 3.8%) than the yellow controls (22.1 ± 4.3%) (t = 4.97, df = 22, *p* < 0.001). There were no differences in trap capture between the different black and yellow patterns despite the differences in edge lengths (H = 3.81, df = 3, *p* = 0.283, Table 2). Of the psyllids collected on these patterned cards, 50 ± 3% were found on the yellow portion. For each black pattern, there were no differences between numbers of psyllids collected on the yellow or black surfaces (α = 0.05).

#### 3.1.3. UV Reflectance or Fluorescence

**Figure 3:**
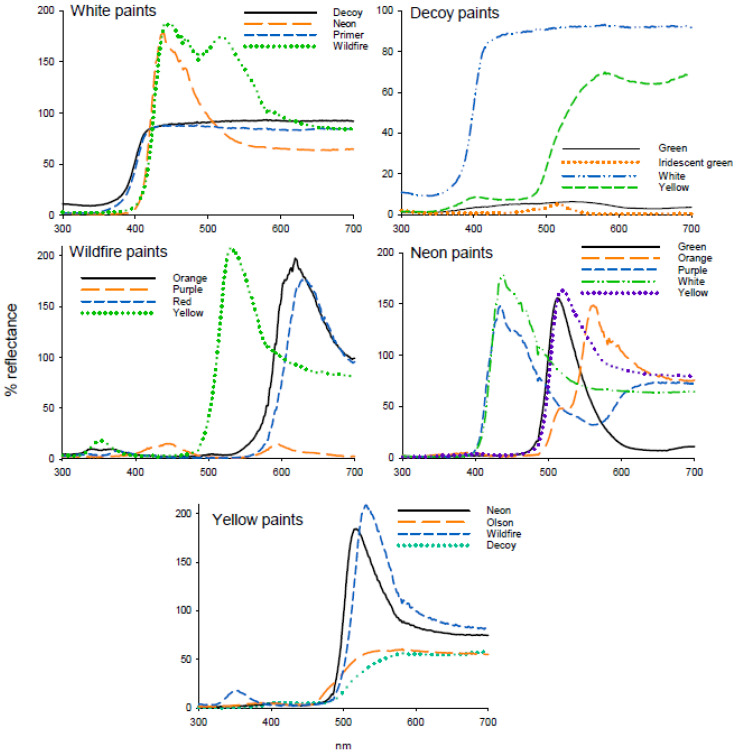
Reflectance spectra of paints used in attraction studies.

In experiment 1, there was no difference in attraction to white paints that differed in reflectance (F = 0.51; df = 3, 47; *p* = 0.677) (Table 3). Reflectance from the white titanium dioxide primer was negligible in the UV (300–700 nm), with high reflectance in the visible spectrum (400–700 nm) (Figure 3). The decoy white reflectance spectrum was similar in shape but with low reflectance in UV (300–400 nm) and slightly higher reflectance from 500 to 700 nm. Both the Wildfire and neon paints contained fluorescent pigments and had low reflectance up to 416 nm. Wildfire white paint had a major peak at 446 nm (187%) and a secondary peak at 517 nm (174%) while neon white paint had one peak at 438 nm (178%). As reflectance was standardized to a nonfluorescent UV-reflecting white standard, peaks for fluorescent paints were greater than 100% (Figure 3).

In experiment 2, attraction of adult *D. citri* differed among decoy paint colors (H = 23.04; df = 3; *p* < 0.001), with yellow the most attractive color, followed by green and iridescent green, and the lowest attraction to white (Table 3). Decoy white had the highest reflectance (92%), with low reflectance from green (6.3%) and iridescent green (5%) and moderate reflectance from decoy yellow paint (69.3%) (Figure 3). Within the comparison of Wildfire fluorescent paints in experiment 3, attraction of *D. citri* differed between paint colors (F = 25.03; df = 3, 47; *p* < 0.001), with the greatest percentage of psyllids captured on orange and yellow sticky traps compared with red and purple (Table 3). Reflectance of all Wildfire paints was low under 580 nm other than a small peak of yellow and orange paints in the UV (352 nm, 18.0 and 9.3%, respectively) and a peak in the blue region (447 nm, 15%) for red paint (Figure 3). Yellow paint had a high peak of reflectance at 531 nm (209%), orange at 619 nm (197%) and red at 627 nm (176%).

Within the comparison of neon fluorescent paints in experiment 4, attraction differed between paints (F = 26.84; df = 4, 59; *p* < 0.001). Attraction was greatest to green, followed by orange and yellow with the lowest attraction to violet (Table 3). White had the highest reflectance at 438 nm (178%), with similar and high reflectance from purple (435 nm, 162%), green (512 nm, 155.6%), yellow (517 nm, 184%) and orange paints (558 nm, 146%) (Figure 3). In experiment 5, attraction to yellow paints differed significantly (F = 3.371; df = 3, 79; *p* < 0.023), with decoy yellow being significantly more attractive than the other yellow paints tested (Table 3). Reflectance of neon and Wildfire yellow was very similar with large peaks at from 520 to 540 nm. Decoy and Olson yellow were similar in shape except for the UV reflectance peak and the higher reflectance from 480 to 580 nm for Olson yellow (Figure 3)

**Figure 4:**
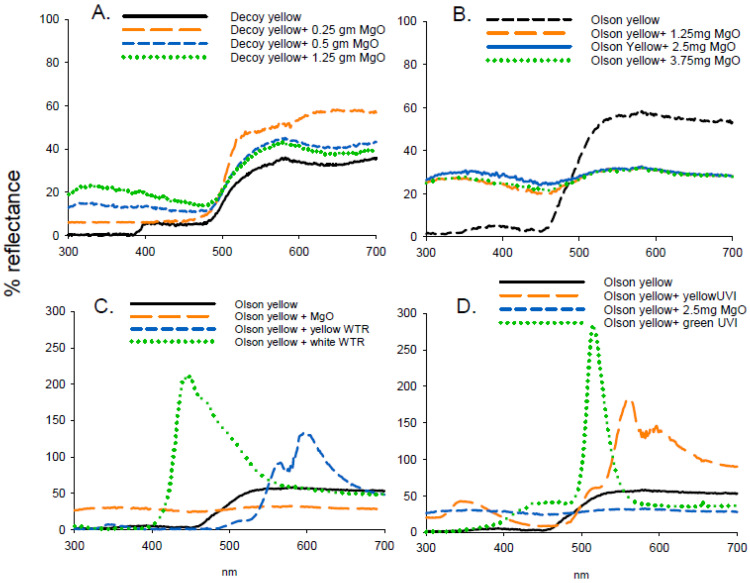
Reflectance spectra from decoy or Olson yellow traps in conjunction with various UV-reflecting (magnesium oxide (MgO)) or fluorescent (WTR, UVI) pigments including: (**A**). Decoy yellow and MgO, (**B**). Olson yellow and MgO, (**C**). Olson yellow with MgO or WTR pigments, and (**D**). Olson yellow in conjunction with MgO and UVI pigments.

Reflectance of MgO was high (>96%) between 300 and 700 nm (Figure 4) and similar to that reported by George et al. [9]. In experiment 6, the addition of MgO (1.25 to 3.75 g) did not enhance attraction to Olson yellow traps (F = 1.162; df = 3, 47; *p* = 0.335) (Table 4). The white WTR fluorescent powder had a large fluorescent peak at 447 nm of over 200% and at 600 nm of 125%, respectively (Figure 4). Addition of MgO increased UV reflection by 27% and reduced reflectance from 480 to 700 nm by 25% compared to Olson yellow traps. In experiment 7, there were significant differences with additions of WTR pigments to Olson yellow (F = 10.901; df = 3, 47; *p* < 0.001), with the highest attraction to Olson yellow combined with yellow WTR and Olson yellow alone. The combination of Olson yellow with MgO was less attractive than WTR pigment but similar to Olson yellow alone. The least attractive combination was Olson yellow with white WTR pigment (Table 4). Green UVI fluorescent pigment had highest reflectance at 514 nm (284%) and yellow UVI pigment peaked at both 343 nm (43%) and 558 nm (182%). In experiment 8, there were significant differences between traps evaluated (F = 66.25; df = 3, 47; *p* < 0.001) (Table 4), with the greatest attraction to Olson yellow, followed by Olson yellow with MgO with the lowest attraction to Olson yellow in conjunction with yellow or green UVI pigment (Table 4).

#### 3.1.4. Paint Mixtures

**Figure 5:**
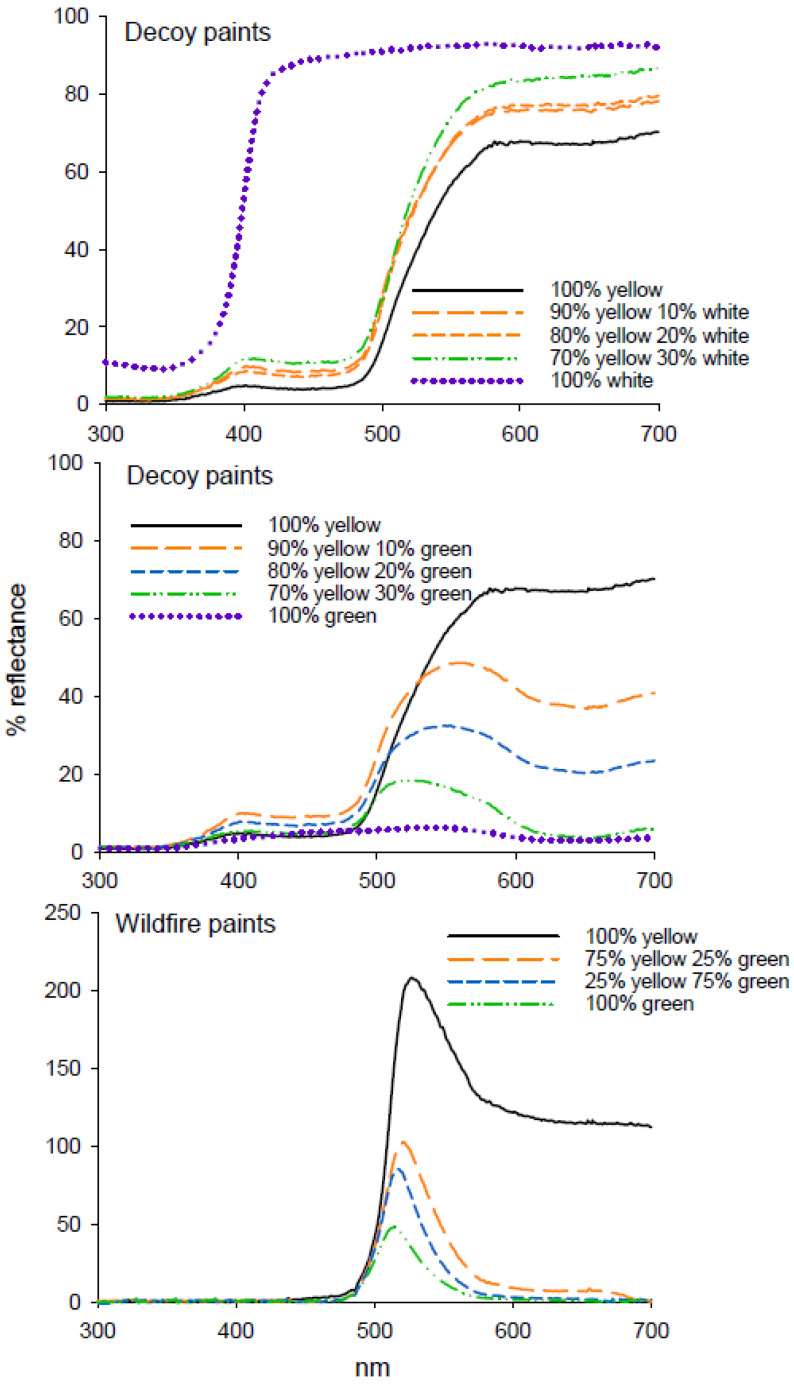
Reflectance spectra from decoy and Wildfire paints in mixtures of either white or green as used in attraction assays of *D. citri*.

Addition of white decoy paint to yellow decoy paint resulted in a 28% increase in reflectance from 575 to 700 nm (Figure 5); however, this increase in intensity did not result in an increase in *D. citri* catch in experiment 9 (H = 3.6.86, df = 3, *p* = 0.297) (Table 5). The addition of decoy yellow to decoy green increased the intensity of the green paint mixtures as well as shifting the wavelength of maximum reflectance towards higher wavelengths (Figure 5), and all paint mixtures containing yellow were more attractive to *D. citri* than 100% green (F = 3.904, df = 3, 47; *p* = 0.007) (Table 5) in experiment 10. Addition of yellow fluorescent Wildfire paint to green Wildfire paint increased reflectance (Figure 5), but did not increase attraction of *D. citri* to traps (F = 1.799, df = 3, 47; *p* = 0.61) (Table 5) in experiment 11.

### 3.2. Field Data

**Figure 6:**
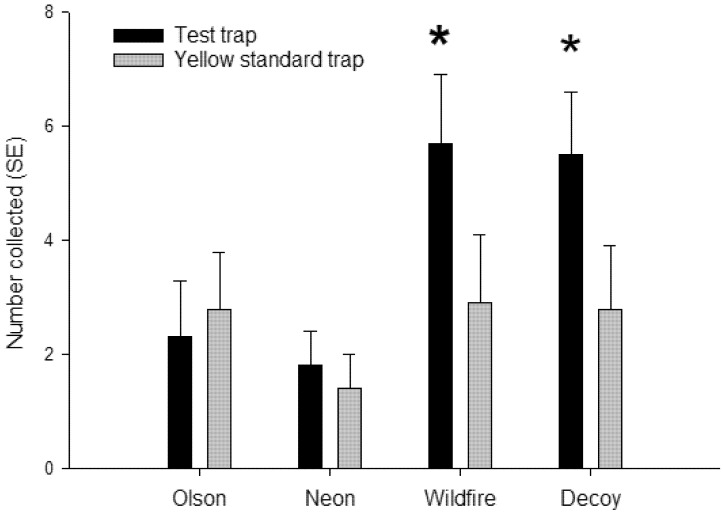
Mean ± SEM capture of adult *D. citri* on adhesive traps deployed in a Florida citrus grove (*n* = 12). Traps consisted of two fluorescent yellows (Wildfire, neon)—a standard yellow trap (Olson) and a UV-reflecting decoy yellow trap. Each trap was paired with a standard yellow trap. * represent significant differences (α = 0.05, paired *t*-test) in trap catch compared to a paired standard yellow sticky trap.

Fluorescent Wildfire sticky traps and decoy yellow sticky traps caught significantly more psyllids than their paired standard yellow sticky cards (*t* = 2.34; df = 1, 11; *p* = 0.02; *t* = 2.56; df = 1, 11; *p* = 0.03; respectively) (Figure 6). In comparison, there were no differences in psyllid captures between fluorescent neon or Olson yellow and their paired standard yellow sticky traps (*t* = 0.65; df = 1, 11; *p* = 0.53; *t* = 0.48; df = 1, 11; *p* = 0.64; respectively). Collections on the the most attractive yellow traps were twice as high as that on the standard yellow traps. Overall, the treatment sticky cards attracted more psyllids than the standard yellow sticky cards (*p* = 0.01, *n* = 48).

## 4. Discussion

Orientation of adult *D. citri* to visual cues is the basis for all commercially available traps for this species. Numerous studies have documented the use of yellow or yellow/green traps for this purpose [6,7,13]. The studies presented here evaluated attributes of visual targets for attraction and capture of *D. citri*. A UV-reflecting yellow (decoy yellow) was more attractive than a commercial standard, the Olson yellow sticky trap in the laboratory and field comparisons.

Addition of LEDs has been shown to increase trap catch of a variety of insect species [38,39,40,41,42]. Previous use of white LEDs has been to enhance collections of adult *D. citri* on yellow sticky traps placed in dark shipping containers [43]. In laboratory evaluations, equivalent attraction of *D. citri* was demonstrated to white and green LEDs [13] or to white, UV, yellow and green LEDs [17]. In our study, UV violet and white LEDs increased trap catch of *D. citri* on yellow sticky cards by 16.0% and 9.8%, respectively. In contrast, addition of yellow LEDs did not increase trap catch. Increased psyllid attraction to UV violet LEDs observed here was likely caused by wavelengths of light similar to that observed by Paris et al. [17]. Increased attraction due to white LEDs is presumed to be the result of enhanced apparency of the trap with a light source. Use of a yellow LED against a yellow trap may not have provided sufficient contrast to significantly impact attraction. The increased attraction to traps observed in the laboratory caused by white or UV violet LEDs warrants further evaluation under field conditions.

Patterns with contrasting edges are considered to have a stimulatory effect on compound eyes and serve as the basis for potential attraction [44]. High-contrast edges can facilitate detection of visual targets by insects, as shown with stored product beetles [45] and whiteflies [23], in which vertical black stimuli elicited attraction behavior. Black patterns have also been reported to increase house fly attraction to sticky traps [33,34]. Printed black patterns on yellow backgrounds increased whitefly (*T. vaporariorum*) collection by 1.4- to 2.3-fold on targets in field studies [23]. Edge complexity may not necessarily enhance attraction, as the contrast of a larger solid object against a background may play a greater role than pattern detail [46]. For *D. citri*, addition of black edges to yellow traps enhanced collection by approximately 2-fold, presumably through enhanced apparency of the traps. There were no differences in attraction; however, between different black patterns despite increased complexity or lengths of edges. The increased collections with black edges on yellow traps in the laboratory setting is promising and will be evaluated under field conditions in the future.

Responses of *D. citri* to white traps [16] as well as white LEDs [17] are generally low. However, with the recent discovery that UV reflectance plays an important role in attraction to specific colors such as yellow or green [9,18], the potential for enhancing attraction to white through use of UV reflection was examined with white decoy paint. Additionally, the enhanced reflectance or intensity from white surfaces was examined with use of fluorescent white paint. However, neither the addition of UV reflectance or white fluorescence affected *D. citri* response to white surfaces, further underscoring the import role of hue in attraction of this species.

Fluorescence is the absorption of short-wavelength radiation and emission at longer wavelengths [47], resulting in the increased intensity of light in the portion of the spectrum where light is emitted or reflected and decreased from the portion where light is absorbed. One requirement for fluorescence to play a role in insect attraction is that the spectral sensitivity of the insect is attuned to those wavelengths [48]. Fluorescence can serve to greatly heighten contrast against the background [47]. While it can play a role in animal biocommunication [49,50], it is less documented in insects. Several studies have documented insect attraction to fluorescent surfaces [48,51,52,53,54] including attraction of some thrips species to fluorescent yellow targets [55]. While plants may contain various fluorescent molecules [56], the contribution of fluorescence to host plant location is considered mild compared to reflected colors [57]. Increased response to fluorescence is not universal and its presence may not affect attraction [57,58,59,60]. While yellow fluorescence has been demonstrated in yellow flowers [61], we had not predicted that this aspect of the visual cue would impact response of *D. citri*. Instead, we postulated that the addition of yellow fluorescent pigment might enhance known attraction of *D. citri* to a portion of the visual spectrum related to yellow-green leaf flush similar to the supernormal stimulus proposed by Prokopy and Owens [20]. In the current study, addition of white fluorescent pigment did not enhance attraction of *D. citri* despite greatly increasing reflectance and thus the intensity of light from the painted surfaces. Orange and yellow Wildfire fluorescent paints were more attractive than red or purple paints, confirming prior results indicating strong preference of *D. citri* to yellow [7,16]. For neon fluorescent paints, the greatest attraction was to a bright green, followed by yellow and orange, with lowest responses to purple and white. Addition of yellow fluorescent WTR pigment did not enhance attraction to Olson yellow traps and addition of WTR fluorescent pigment reduced attraction, possibly due to obscuring yellow reflectance from the traps. Similarly, addition of fluorescent yellow or green UVI pigment reduced attraction to the traps, possibly because yellow reflectance from the traps was obscured.

Iridescence or the structural coloration that differs with angle of view has been reported as increasing detectability by bumblebees by enhancing contrast against the background [24]. While iridescence has been little studied in terms of insect attraction, a commercially available paint providing iridescent pigment was evaluated. Use of a paint with iridescent pigment did not enhance attraction of psyllids. The reflectance of this paint measured here was low and thus the corresponding low level of psyllid attraction it caused is not surprising.

Traps reflective in yellow wavelengths (560–590 nm) are highly attractive to many plant-feeding insects, especially Hemiptera [20,21,62]. For instance, some species of alate aphids preferentially land in response to wavelengths representing yellow/green [22,63]. Attributes of yellow such as wavelength and intensity may differ such that attraction to the traps varies. For example, in a comparison of different colored traps, including a fluorescent and non-fluorescent yellow, captures of two psyllid species that feed on the host plant, *Eucalyptus globulus*, were greater on fluorescent yellow traps [64]. However, two other psyllid species preferred red stimuli, which corresponded to coloration of young anthocyanic eucalyptus leaves [65]. Hall et al. [7] compared attraction of *D. citri* to different yellow and yellow/green traps in citrus orchards in Florida and Texas. While there were seasonal differences, not all yellow traps were equally attractive. In that study, trap catch of adults increased with higher proportions of yellow, orange and red wavelengths but decreased with an increase in proportion of blue wavelengths [7]. This strong attraction to yellow/green is presumed to be associated with yellow/green color of flush [19]. Blue traps have previously been shown to be unattractive to *D. citri* [66]. While Olson and decoy yellow were relatively similar in reflectance in this study, the higher reflectance of blue (450–490 nm) and possibly green (490–560 nm) of the Olson yellow compared to the decoy yellow may explain the higher attraction of *D. citri* to decoy yellow. A similar conclusion with *Rhagoletis cerasi* (L.) was made in that yellow with increased reflection of blue were less attractive [67]. In our field evaluation of yellow traps, the decoy yellow traps were more attractive to *D. citri* than the Olson yellow traps. In the laboratory, both fluorescent yellows (Wildfire, neon) were less attractive compared with decoy yellow. However, in field evaluations, the Wildfire yellow was as attractive as the decoy yellow. Differences in lighting between the laboratory and field, and possible higher reflectance of green and yellow from the Wildfire yellow compared to neon yellow may have contributed to these differing results. The interaction between yellow and blue reflectance on psyllid behavior suggests a role of central processing of these visual signals from the blue and yellow photoreceptors of *D. citri*. Future research is warranted to examine this relationship in visual attraction of *D. citri* and may help refine field traps for *D. citri*.

The intensity or brightness of a visual target can enhance its apparency, either through chromatic or achromatic means [32], with contrast against a background [29,30,45] possibly contributing to increased attraction to the target. Enhancement of target intensity or reflectance resulted in increased attraction of thrips [68,69]. While decoy yellow was highly attractive to *D. citri*, addition of white pigment increased reflectance but did not increase attraction. Similarly, addition of green pigment to either decoy or Wildfire paints did not enhance attraction. Citrus species with higher overall reflectance such as lemon are preferred by *D. citri*, as are emergent leaves (flush) that are characteristically yellow/green and high in reflectance [16,18]. As the increased proportion of white pigment increased reflection, it also decreased saturation of the yellow pigment. The unaltered decoy yellow provided the highest level of attraction.

Reflected UV light has been shown to play a role in host plant location and orientation during insect flight [40]. Reflectance of UV from surfaces impacts landing responses [70,71] and high reflectance of UV from mulches reduced landing and infestation by thrips [72] and *D. citri* [73]. In contrast, lower-intensity UV-emitting sources such as LEDs have been reported as attractive for a range of insect species [17,74,75]. Adult *D. citri* were more attracted to UV, yellow and green LEDs than blue LEDs [17]. Wavelength-associated phototaxis occuring under illumination is greater toward sources of shorter (350–405 nm) than longer (500–620 nm) wavelength light [18]. The role of UV was further elucidated by Paris et al. [19], who reported that removal of UV components of yellow or green light reflected from surfaces reduced attraction of *D. citri,* indicating the combination of both UV and visible spectrum wavelengths played a role in attraction. Similarly, removal of UV wavelengths from light illuminating crops through use of UV absorbing film or screens reduced insect infestations [71,76] and reduced take-off and host-finding behavior of *D. citri* [77]. The concept of combinating UV and yellow as an attractant for bees was termed “bee purple” by Daumer [78] and was also demonstrated as important in attraction of *Heliconius erato* scales [61]. Addition of a UV-reflecting pigment, magnesium oxide, to decoy yellow targets enhanced attraction of *D. citri* [9]. When magnesium oxide was added to phagostimulant blends of chemicals, attraction and probing by *D. citri* were increased [9]. This increased attraction was associated with increased UV reflectance from the visual targets. In contrast, in the current study, addition of magnesium oxide did not increase attraction to the Olson yellow sticky cards. The differences in enhancement of attraction through addition of a UV-reflecting pigment, magnesium oxide, are likely related to the differences in attraction to the yellow targets, as the decoy yellow targets are intrinsically more attractive to *D. citri* than standard Olson yellow targets.

## 5. Conclusions

We have shown that several aspects of Olson yellow sticky visual traps can be altered to increase attraction of adult *D. citri* in the laboratory. Addition of black edges increased collection of psyllids. However, there were no differences between black patterns. Use of a UV-reflecting yellow paint increased psyllid attraction, as compared with standard yellow sticky traps in laboratory and field comparisons. Additionally, white or UV violet LEDs enhanced attraction of *D. citri* to yellow visual traps in the laboratory. These additions appear promising and with future evaluations, under orchard conditions, could provide improved tools for more sensitive surveillance of the Asian citrus psyllid.

## Figures and Tables

**Table 1:** Mean percent ± SEM capture (%) of adult *D. citri* in paired tests on Olson yellow sticky traps with and without an attached LED in laboratory cages (*n* = 20).

LED	% Capture	*p*	*t*-Value (df)
None	41.9 ± 3.3 a	0.002	3.39 (38)
UV violet	58.1 ± 3.3 b		
None	45.6 ± 3.3 a	0.024	2.34 (38)
White	55.4 ± 3.3 b		
None	46.4 ± 4.2 a	0.07	1.48 (38)
Yellow	54.2 ± 4.2 a		
White	49.9 ± 3.5 a	0.959	0.052 (38)
UV violet	50.1 ± 3.5 a		

Within each paired comparison, means followed by different letters are significantly different (α = 0.05, paired *t*-test).

**Table 2:** Mean ± SEM capture (%) of adult *D. citri* on sticky traps with one of four patterns of yellow and black surfaces deployed simultaneously in laboratory cages (*n* = 12). Half of the surface area of each trap was yellow.

Pattern	Black/Yellow Edge Length (cm)	Capture (%)
Single large square	28.72	27.6 ± 2.0
Wide stripes	50.8	25.0 ± 1.5
Narrow stripes	111.76	23.9 ± 1.8
Small squares	85.92	23.8 ± 2.2

Means were not significantly different (α = 0.05, Kruskal–Wallis-ranked ANOVA).

**Table 3:** Mean ± SEM capture (%) of adult *D. citri* on sticky traps in multi-choice assays in laboratory cages (*n* = 12).

Experiment	Paint	Capture (%)
1. White paints	Decoy	25.8 ± 2.6 a
	Neon	22.5 ± 2.4 a
	Primer	27.2 ± 2.6 a
	Wildfire	24.5 ± 3.3 a
2. Decoy paints	Green	25.2 ± 3.0 b
	Iridescent green	15.3 ± 2.6 bc
	White	15.3 ± 3.3 c
	Yellow	44.2 ± 5.3 a
3. Wildfire paints	Orange	38.5 ± 3.0 a
	Purple	9.3 ± 1.8 b
	Red	17.1 ± 1.9 b
	Yellow	35.1 ± 3.9 a
4. Neon paints	Green	35.5 ± 2.4 a
	Orange	26.0 ± 3.0 b
	Purple	6.5 ± 1.8 c
	White	6.3 ± 1.8 c
	Yellow	25.7 ± 3.1 b
5. Yellow paints	Decoy	31.1 ± 2.7 a
	Neon	23.1 ± 1.8 b
	Olson	24.5 ± 2.3 b
	Wildfire	21.3 ± 2.4 b

Within each experiment, means followed by different letters are significantly different (α = 0.05, Tukey’s HSD following a significant ANOVA).

**Table 4:** Mean ± SEM capture (%) of adult *D. citri* on Olson yellow traps augmented with UV-reflecting (magnesium oxide (MgO)) or fluorescent (WTR, UVI) pigments in laboratory cages (*n* = 12).

Experiment	Additional Pigments	Capture (%)
6. UV reflecting	None	28.7 ± 2.9 a
	1.25 gm MgO	25.2 ± 2.2 a
	2.50 gm MgO	23.7 ± 2.5 a
	3.75 gm MgO	22.4 ± 2.5 a
7. Fluorescent WTR	None	29.5 ± 2.5 ab
	2.5 gm MgO	22.9 ± 2.7 b
	2.5 gm yellow WTR	34.1 ± 3.4 a
	2.5 gm white WTR	13.5 ± 2.9 c
8. Fluorescent UVI	None	49.6 ± 2.7 a
	2.5 gm MgO	23.6 ± 2.7 b
	2.5 gm yellow UVI	12.8 ± 1.2 c
	2.5 gm green UVI	13.9 ± 1.3 c

Within each experiment, means followed by different letters are significantly different (α = 0.05, Tukey’s multiple range test).

**Table 5:** Mean ± SEM capture (%) of adult *D. citri* on sticky traps coated with different paint mixtures in multi-choice assays in laboratory ages (*n* = 12).

Experiment	Mixture		Capture (%)
	**% Yellow**	**% White**	
9. Decoy	100	0	20.6 ± 2.6 a
	90	10	21.7 ± 3.0 a
	80	20	28.5 ± 3.5 a
	70	30	29.2 ± 3.9 a
	% yellow	% green	
10. Decoy	100	0	20.2 ± 2.5 a
	90	10	24.0 ± 2.9 a
	80	20	22.8 ± 3.0 a
	70	30	21.9 ± 2.9 a
	0	100	11.1 ± 1.5 b
	% yellow	% green	
11. Wildfire	100	0	30.9 ± 3.8 a
	75	25	25.9 ± 4.0 a
	25	75	23.8 ± 3.0 a
	0	100	19.3 ± 3.4 a

Within each experiment, means followed by different letters are significantly different (α = 0.05, Mann–Whitney *U* test).
